# Determinants of Cervical Cancer Screening Among Rural Women in Zimbabwe

**DOI:** 10.1111/phn.13490

**Published:** 2024-11-15

**Authors:** Paddington T. Mundagowa, Oscar Tapera, Bothwell Guzha, Megan Burke Fitzpatrick, Racheal S. Dube Mandishora, Mufaro Kanyangarara

**Affiliations:** ^1^ Department of Epidemiology and Biostatistics, Arnold School of Public Health University of South Carolina Columbia South Carolina USA; ^2^ Sadtap Health Research Institute (SHRI) Harare Zimbabwe; ^3^ Department of Obstetrics and Gynecology University of Zimbabwe Faculty of Medicine and Health Sciences Harare Zimbabwe; ^4^ Department of Pathology and Laboratory Medicine University of Wisconsin School of Medicine and Public Health Madison Wisconsin USA; ^5^ Department of Laboratory Diagnostic and Investigative Sciences, Medical Microbiology Unit University of Zimbabwe Faculty of Medicine and Health Sciences Harare Zimbabwe; ^6^ Center for Immunization and Infection Research in Cancer Moffitt Cancer Center Tampa Florida USA

**Keywords:** cervical cancer screening, human papillomavirus, rural, sub‐Saharan Africa

## Abstract

**Objective:**

To identify the determinants of cervical cancer (CC) screening among underserved rural women in Zimbabwe.

**Design:**

Community‐based cross‐sectional survey.

**Sample:**

840 rural women (25–65 years).

**Measurements:**

A structured, pretested questionnaire was used to collect data on sociodemographic characteristics and factors influencing screening. The outcome was defined as self‐reported ever screening for CC. Multivariable logistic regression was used to examine the association between CC screening and independent variables.

**Results:**

Of the 840 women included, 33% had a history of screening. Women 25–45 years (adjusted odds ratio (aOR): 0.43; 95% CI: 0.30–0.61) and without medical insurance (aOR: 0.66; 95% CI: 0.45–0.97) had reduced odds of CC screening. Conversely, women who had seen or heard messages on CC screening (aOR: 1.48; 95% CI: 1.03–2.13), were living with HIV (aOR: 1.87; 95% CI: 1.22–2.87) reported recent antibiotic use (aOR: 4.50; 95% CI: 1.47–13.79) and had malaria in the last 6 months (aOR: 2.45; 95% CI: 1.02–5.86) had increased odds of CC screening.

**Conclusion:**

There is a need for intensified efforts to improve CC screening uptake, particularly in underserved rural areas with suboptimal screening rates and high CC burden. Strategies should include widespread tailored awareness messaging targeting younger women, women living with HIV, and women without medical insurance.

**Abbreviations**
CC: cervical cancer
CHW: community health worker
HIV: human immuno‐deficiency virus
HPV: human papillomavirus
LMICs: low‐and middle‐income countries
VIAC: visual inspection with acetic acid and cervicography
WHO: World Health Organization

## 1 Background

Human Papillomavirus (HPV) is the most common sexually transmitted infection, affecting mucosal and cutaneous epithelia (Gheit 2019). However, most (>90%) genital HPV infections resolve naturally within 2 years (Maucort‐Boulch et al. 2010), persistent infections can arise due to various factors such as age, cigarette smoking, number of sexual partners, genetic dispositions, physical well‐being, and weakened immune responses, especially HIV infection (Bray et al. 2018). Persistent infections increase the risk of cancer, including anal, vulvar, vaginal, penile, head and neck, and CC.

The World Health Organization (WHO) advocates for CC screening coverage to reach 70% by the year 2030 and recommends that screening should be done at least twice in a woman's lifetime by the ages of 35 and 45 using high‐performance DNA‐based tests if cervical cancer is to be eliminated as a public health problem by the year 2120 (World Health Organization 2021). In addition, 90% of eligible girls should receive the HPV vaccine, and optimal treatment for 90% of women with precancer and invasive CC (World Health Organization 2021). Treatment of precancerous lesions can be ablative, using mostly thermal ablation or cryotherapy or excisional through loop excision of the transformation zone (LLETZ), cone biopsy, and in some cases, a simple hysterectomy (World Health Organization 2024a). Scaling up HPV vaccination in low‐ and middle‐income countries (LMICs) is projected to reduce the median age‐standardized CC incidence by 89.4% and prevent 61 million cases over the next century (Brisson et al. 2020). HPV screening is anticipated to decrease CC incidence by 96.7% and prevent 12.1 million cases over the same period (Brisson et al. 2020). Increasing screening using HPV testing will expedite the decline in CC cases and is poised to eliminate the disease in high‐burden countries by 2120 (Brisson et al. 2020).

Cervical cancer represents a significant public health burden, with an estimated 660,000 new cases and 350,000 deaths recorded in 2022 alone (World Health Organization 2024a). It ranks as the fourth most common cancer among women globally, disproportionately affecting women in poor, underserved, and rural settings (Beavis, Gravitt, and Rositch 2017; de Sanjosé et al. 2007; Sung et al. 2021). More than 80% of CC cases occur in LMICs, with the sub‐Saharan Africa region accounting for the highest incidence and mortality rates (Arbyn et al. 2020).

In Zimbabwe, CC accounts for one‐third of all cancers affecting women, further compounded by one of the world's highest HIV prevalence rates (15.3% among women) (Ministry of Health and Child Care 2021). HIV‐co‐infected women exhibit increased susceptibility to HPV infection due to compromised immune systems (Tagne Simo et al. 2021). In Zimbabwe, 67.9% of invasive CC cases are attributed to HPV 16/18 (Chin'ombe et al. 2014). Approximately 3,043 women are diagnosed with CC annually, and an estimated 1,976 succumb to the disease every year (ICO/IARC HPV Information Center 2023). The annual national incidence of CC is 61 per 100,000 women (World Health Organization Zimbabwe 2023). In 2013, Zimbabwe adopted visual inspection with acetic acid and cervicography (VIAC) as the recommended CC screening method in the public sector. VIAC is still the primary screening method in the country because it is relatively inexpensive and provides immediate results, allowing for a “see and treat” approach where treatment can be administered right after a positive screening (Tachiwenyika et al. 2023; Tapera et al. 2021). The WHO recommends the “screen‐and‐treat” approach using VIAC as an effective, low‐cost strategy to reduce cervical cancer incidence in LMICs (Tachiwenyika et al. 2023). To improve the overall effectiveness of the CC screening process and prompt intervention, Zimbabwe currently has over 60 sites where HPV testing is performed in conjunction with VIAC (World Health Organization 2024b). Despite the cost‐effectiveness of visual inspection with VIAC for CC screening (Kessler 2017), uptake remains alarmingly low, especially among rural women. A recent nationwide survey revealed a CC screening rate of 27.8 (95% CI: 26.7–29.4) (Mundagowa, Liu, and Kanyangarara 2024).

Rural women face significant geographical, socioeconomic, and cultural barriers in accessing CC screening information and services, leading to poor health‐seeking behavior (Vanderpool, Stradtman, and Brandt 2019; Zhetpisbayeva et al. 2023). Most rural areas have chronic challenges regarding access to health services due to lack of transport and long traveling distances to the nearest health center. Women living in rural areas often lack autonomous decision‐making in a patriarchal society, stigma around reproductive health issues and cancer, male providers, reduced perceived threat of CC, and poverty (Fitzpatrick et al. 2020; Vanderpool, Stradtman, and Brandt 2019; Zhetpisbayeva et al. 2023). The reasons behind the geographic disparities in CC distribution are complex and require setting‐specific investigation.

Although contemporary research has advanced our understanding of CC epidemiology, risk factors, causes, and preventive measures, disseminating this knowledge in LMICs has been slow and poorly implemented. For instance, in 2015, 79% of Zimbabwean women reported hearing about CC, yet only 13% had ever been screened (Zimbabwe National Statistics Agency and ICF International and Zimbabwe National Statistics Agency ‐ZIMSTAT 2016). Despite numerous awareness campaigns, CC screening uptake in sub‐Saharan Africa remains low, primarily due to insufficient knowledge and prevalent misinformation surrounding the disease and screening procedures (Gwavu, Murray, and Okafor 2023; Jedy‐Agba et al. 2020; Lim and Ojo 2017). The Ministry of Health and Child Care of Zimbabwe is working towards improving access to screening and early treatment.

A review of CC knowledge in Zimbabwe revealed misconceptions regarding its causes and treatment (Kuguyo et al. 2017). CC was erroneously perceived as uterine dirt caused by sperm retention, insertion of vaginal preparations (e.g., herbs), multiple sexual partners, cold weather, and witchcraft (Kuguyo et al. 2017). There is limited context‐specific and community‐based data on the individual and community‐level determinants of CC screening opportunities and most data is from facility‐based surveys in Zimbabwe. To address this evidence gap and inform interventions that enhance awareness and education efforts, it is critical to understand the characteristics of women who participate in CC screening programs based on community surveys. This study aimed to identify the determinants of CC screening among rural women in Zimbabwe, a critical step toward developing evidence‐informed prevention, screening, and early treatment strategies for women at the highest risk of CC in rural sub‐Saharan Africa. Guided by the determinants of CC screening in rural areas, program managers can implement potential interventions to improve screening and treatment services using community engagement and multi‐sectoral collaborations.

## 2 Methods

### 2.1 Study Design and Setting

This study constitutes a secondary analysis of data collected from a community‐based cross‐sectional survey conducted in Hurungwe District, Zimbabwe, as part of a randomized clinical trial (RCT). This setting was selected because of its low CC screening coverage, poor access to health services, and convenience to the researchers due to prior working relationships with health authorities in the area—the trial aimed to evaluate the effectiveness of community‐based HPV‐self‐sampling in improving CC screening participation in rural Zimbabwe. Hurungwe is a rural district in the northern part of the country, with about 89,117 households and an estimated population of 390,907, of which women comprise 50.1% of the population (Zimbabwe National Statistics Agency‐ZIMSTAT, 2022). The study was conducted in nine randomly selected villages in the Chidamoyo area, encompassing administrative wards 13 and 15, with an approximate population of 32,000 (Fitzpatrick et al. 2019). Chidamoyo Christian Hospital, a 100‐bed rural health facility with 18 outreach clinics, provides primary health services to more than 70,000 patients annually, predominantly poor local subsistence farmers in the area. The hospital's operational costs are primarily covered by donor funding. We previously conducted a CC self‐sampling study in some of the communities in 2017, at which time the baseline screening was only approximately 5%. More than 600 women were screened during the 2017 study.

### 2.2 Sample Size

The sample size was 1,017 women, with 113 women from each of the nine villages. The minimum sample size required was based on detecting differences of 10% (unadjusted) and 35% (standardized) in participation rates in the intervention. Calculations were conducted using the National Institutes of Health (NIH) sample size calculator (National Institute of Health 2023), assuming a participation rate of 5% in the control arm, a type I error of 5%, a power of 80%, and an intercluster correlation coefficient of 0.0044 (Hade et al. 2010).

### 2.3 Study Population

The study population comprised women aged between 25 and 65 years who resided in the selected villages in Hurungwe District. Women who had undergone a hysterectomy, those under the age of 25, and those with missing outcome data were excluded from the present analysis. The exclusion of individuals under the age of 25 was in line with WHO recommendations for CC screening to commence at the age of 25 at the time the study was conducted (World Health Organization 2021).

### 2.4 Data Collection

The villages were randomized using the ballot approach because they had similar sociodemographic characteristics. Three Community Health Workers (CHWs) were assigned to pick three villages (clusters) each from a ballot of all Chidamoyo villages. The sampling frame was a list of all women aged 25–65 years from the selected villages compiled by the CHWs. All eligible women were approached for informed consent before the interview. By including all women who meet the inclusion criteria, we ensured an equal chance of participation among eligible women.

Trained CHWs approached women in selected villages and administered a structured, pretested questionnaire. The questionnaire collected data on participants’ sociodemographic characteristics, knowledge, attitudes, and practices related to CC. Data collection occurred between March and June 2021.

### 2.5 Study Variables

The primary outcome variable was ever having been screened for CC using either a PAP smear or VIAC. The WHO recommends that the general population of women get screened for CC starting at the age of 30 years, while women living with HIV can start screening at 25 years (World Health Organization 2021). A lower limit of 25 years for screening was used in this study due to the high HIV prevalence in the study population. Exposure variables included sociodemographic and health characteristics, knowledge, attitudes, and practices. Age was retained as a continuous variable. Marital status (married/cohabiting vs. not married/single/divorced/widowed), level of education (grade 7 or lower vs. secondary school or higher), employment status (unemployed vs. formally/self‐employed), household size (<4 vs. ≥4), medical insurance status (no vs. yes), HIV status (negative vs. positive), recent malaria diagnosis (yes vs. no), had bilharzia (yes vs. no), and age at menarche (≤15 vs. >15 years) were treated as dichotomous variables. This cutoff was selected because the average age of menarche among women in sub‐Saharan Africa is 15 years (Garenne 2020). Knowledge was assessed using eight questions covering awareness of messages about CC screening, understanding of CC causes, understanding of the association between CC and HIV, knowledge of the CC problem in Hurungwe, knowledge of service locations, and available services at local facilities. Attitudes were gauged by assessing participants’ perceptions about the stigmatization of CC patients. Practices were assessed by ten questions addressing health‐seeking behavior, HPV vaccination, sexual practices, antibiotic use, smoking habits, and menstrual hygiene.

### 2.6 Statistical Analysis

Data were entered, cleaned, and organized in Microsoft Excel before being imported into the SAS 9.4 (SAS Institute, Cary, NC) statistical package for analysis. Descriptive statistics were presented as frequencies and percentages for categorical data and as means and standard deviations for continuous data. Chi‐square tests were employed to assess differences in sociodemographic and health characteristics, knowledge, attitudes, and practices between participants who had undergone CC screening and those who had not. Univariable and multivariable logistic regression were used to examine associations between screening and exposure variables. Model selection was based on the Akaike Information Criterion (AIC), and the adjusted model with the smallest AIC was used. A test for multicollinearity was tested using the variance inflation factor (VIF), and a VIF of more than 4 was considered multicollinearity requiring correction. Odds ratios and 95% confidence intervals were reported, with statistical significance set at *p* < 0.05.

### 2.7 Ethical Considerations

Ethical approval for the study was obtained from the Medical Research Council of Zimbabwe (MRCZ/A/2621). Written informed consent was obtained from all study participants. Participants were assigned random, unique study numbers, with the key for participant identification stored under secure conditions. Data were stored in a password‐protected computer using unique code identifiers in an encrypted database.

## 3 Results

A total of 1,020 participants participated in the community‐based survey. One participant with missing data on the outcome variable and 179 participants under 25 years were excluded from the analysis. Thus, data from 840 participants were analyzed; of these, 274(32.6%) had a history of CC screening. Table 1 shows the demographic and health characteristics of the participants. The mean age of women who had ever been screened was significantly older (mean: 40.9 ± 8.9 years) compared to women who had not (mean: 36.0 ± 8.7 years). About 45% of the participants were aged 35 or younger, 91% had at least a secondary level education, 81% were married/cohabiting, and 82% did not have medical aid insurance. Approximately 14% were women living with HIV who reported ever screening for CC.

**TABLE 1:** Participant demographic and health characteristics (*n* = 840).

Variables	Characteristics	Ever screened for Cervical Cancer		Chi‐square *p‐* value
		Yes *n* (%)	No *n* (%)	
Age (years)	25–35	80 (21)	300 (79)	<0.01^*^
	36–45	104 (37)	175 (63)	
	46–55	74 (50)	74 (50)	
	56–65	16 (48)	17 (52)	
Mean age (years)		40.9 ± 8.9	36.0 ± 8.7	<0.01^*^
Highest level of education completed	Grade 7 or lower	23 (32)	49 (68)	0.90
	Secondary or higher	251 (33)	517 (67)	
Marital status	Married/cohabiting	210 (31)	471 (69)	0.02^*^
	Not married (single/divorced/widowed)	64 (40)	95 (60)	
Employment status	Unemployed	265 (32)	555 (68)	0.23
	Employed	9 (45)	11 (55)	
Household size (individuals)	<4	167 (30)	383 (70)	0.05^*^
	≥4	107 (37)	183 (63)	
Had medical insurance	No	209 (30)	479 (70)	<0.01^*^
	Yes	65 (43)	87 (57)	
HIV status^a^	Positive	61 (52)	57 (48)	<0.01^*^
	Negative	213 (30)	508 (70)	
Had malaria in the past 6 months	Yes	12 (52)	11 (48)	0.04^*^
	No	262 (32)	555 (68)	
Age at menarche (years)	≤15	191 (32)	397 (68)	0.90
	>15	83 (33)	169 (67)	

Abbreviations: CC, cervical cancer; HIV, human immuno‐deficiency virus.

^a^ *n* = 839.

* *p* value < 0.05.

Table 2 displays the knowledge, attitudes, and practices reported by participants. There was a significant difference in CC screening between participants who had ever heard/seen messages about CC and those who had not (*p* < 0.01). Comparing participants who knew where to get CC screening services and those who did not reveal a significant difference in CC screening uptake. When asked about the CC services offered at the local health facilities, the majority (64%) stated screening services and 64% knew about VIAC as a CC screening method. Only 15% and 1% of the participants correctly identified HPV and HIV as risk factors for CC, and participants did not identify other risk factors such as early marriage, early sexual debut, multiple sexual partners, smoking, poor genital hygiene, and long‐term use of oral contraceptives. About 92% agreed that women with CC should not be stigmatized. There was no significant difference in CC screening between women who had an early sexual debut (under 15 years) and those who had their first sexual encounter at 15 years and older (*p* = 0.52). A significant difference in CC screening was observed in antibiotic use in the last 6 months (*p* < 0.01).

**TABLE 2:** Cervical cancer knowledge, attitudes, and practices of participants.

Variables	Characteristics	Ever screened for Cervical Cancer		Chi‐square *p*‐value
		Yes *n* (%)	No, *n* (%)	
Ever heard/seen messages about CC	Yes	209 (37)	359 (63)	<0.01^*^
	No	65 (24)	207 (76)	
Knows where to get CC services	Yes	243 (35)	455 (65)	<0.01^*^
	No	31 (22)	111 (78)	
CC is associated with HIV	Yes	147 (34)	286 (66)	0.43
	No	98 (33)	203 (67)	
	I don't know	29 (27)	77 (73)	
CC is a major health problem in Hurungwe	Yes	209 (34)	414 (66)	0.23
	No	42 (34)	82 (66)	
	I don't know	23 (25)	70 (75)	
Local health facilities offer CC services^a^	Yes	235 (35)	428 (65)	0.14
	No	3 (43)	4 (57)	
	I don't know	5 (18)	23 (82)	
CC services offered at the local health facility (multiple responses)	Screening	200 (37)	340 (63)	
	Treatment of precancers	33 (39)	51 (61)	
	Diagnosis	18 (25)	54 (75)	
	Treatment of CC	10 (48)	11 (52)	
Methods of CC screening services^a^	VIAC	200 (37)	337 (63)	
	HPV screening	26 (35)	49 (65)	
	I don't know	17 (20)	69 (80)	
Causes of CC (multiple responses)	HPV	52 (41)	74 (59)	
	Using vaginal herbs	182 (36)	327 (64)	
	Using soap to wash the vagina	56 (35)	105 (65)	
	Witchcraft	0 (0)	2 (100)	
	HIV/AIDS	5 (45)	6 (55)	
	I don't know	18 (17)	88 (83)	
	Other	1 (33)	2 (67)	
Women with CC should be stigmatized	Yes	9 (47)	10 (53)	0.20
	No	252 (33)	517 (67)	
	I don't know	13(25)	39(75)	
Vaccinated against HPV	Yes	39 (36)	69 (64)	0.41
	No	235 (32)	497 (68)	
Rinse vagina after sex	Yes	247 (32)	522 (68)	0.31
	No	27 (38)	44 (62)	
Age at sexual debut (years)	<15	17 (37)	29 (63)	0.52
	≥15	257 (32)	537 (68)	
Use lubricants during sex	Yes	3 (43)	4 (57)	0.56
	No	271 (33)	562 (67)	
Used antibiotics in the last 6 months	Yes	11 (69)	5 (31)	<0.01^*^
	No	263 (32)	561 (68)	
Smoke cigarettes	Yes	4 (57)	3 (43)	0.16
	No	270 (32)	563 (68)	
Materials used during the menstrual period	Rag/Cloth	167 (33)	343 (67)	0.34
	Tissue paper	1 (25)	3 (75)	
	Sanitary pads	72 (31)	160 (69)	
	Tampons	1 (100)	0 (0)	
	Cotton wool	39 (26)	111 (74)	
Use herbs in general	Yes	26 (39)	41 (61)	0.26
	No	248 (32)	525 (68)	
Use herbs in the vagina	Yes	8 (31)	18 (69)	0.84
	No	266 (33)	548 (67)	

Abbreviations: CC, cervical cancer; HPV, Human papillomavirus; VIAC, visual inspection with acetic acid and cervicography.

^a^ *n* = 698.

* *p* value < 0.05.

Table 3 presents the findings of the univariable and multiple regression analysis to ascertain the associations between having ever been screened for CC and the exposure variables. Age, medical insurance, seeing or hearing CC screening messages, using antibiotics in the previous 6 months, having had malaria in the previous 6 months, and HIV status were associated with CC screening in the multivariable model. Women 45 years and younger were 67% less likely to have been screened compared to older women (>45 years), while participants who did not have medical insurance were 34% less likely to have been screened for CC. Exposure to CC messages increased the likelihood of being screened by 48% compared to those who were not exposed, and women who had taken antibiotics in the previous 6 months were 3.5 times more likely to be screened compared to those who did not take antibiotics. Getting ill from malaria in the last 6 months was associated with higher odds of being screened (Adjusted odds ratio—aOR: 2.45 [95% CI: 1.02–5.86]). Participants who were living with HIV had higher odds of being screened compared to those who were not living with HIV (aOR: 1.87 [95% CI: 1.22–2.87]). The test for multicollinearity showed that the variable with the highest VIF was 1.2, indicating there was no correlation among the predictors used in the adjusted model.

**TABLE 3:** Results of the univariable and multivariable logistic regression.

Variable	Characteristics	Univariable logistic regression		Multivariable logistic regression	
		cOR (95% CI)	*p*‐value	aOR (95% CI)	*p*‐value
Age (years)	25–45 vs. 45–65	0.37 (0.27–0.50)	<0.01^*^	0.43 (0.30–0.61)	<0.01^*^
Education	Grade 7 or lower vs. secondary or higher	0.97 (0.58–1.62)	0.90	—	—
Marital status	Married/cohabiting vs. not married^*^	0.66 (0.46–0.95)	0.02^*^	0.79 (0.54–1.16)	0.23
Occupation	Unemployed vs. employed	0.58 (0.24–1.43)	0.24	—	—
Household size	<4 vs. ≥4	0.75 (0.55–1.01)	0.06	—	—
Had medical insurance	No vs. yes	0.58 (0.41–0.84)	<0.01^*^	0.66 (0.45–0.97)	0.03^*^
Ever heard/seen messages on CC	Yes vs. no	1.85 (1.34–2.57)	<0.01^*^	1.48 (1.03–2.13)	0.03^*^
Knows where to get CC services	Yes vs. no	1.91 (1.24–2.93)	<0.01^*^	1.43 (0.88–2.31)	0.15
CC is associated with HIV	Yes vs. no	1.14 (0.92–1.40)	0.23	—	—
Age at menarche (years)	≤15 vs. >15	0.98 (0.72–1.34)	0.90	—	—
Age at sexual debut (years)	<15 vs. ≥15	1.23 (0.66–1.27)	0.52	—	—
HIV status	Positive vs. Negative	2.56 (1.72–3.80)	<0.01^*^	1.87 (1.22–2.87)	<0.01^*^
CC is a major health problem in Hurungwe	Yes vs. no	1.18 (0.94–1.47)	0.15	—	—
Use of antibiotics in the last 6 months	Yes vs. no	4.69 (1.61–13.64)	<0.01^*^	4.50 (1.47–13.79)	<0.01^*^
Smoke cigarettes	Yes vs. no	2.78 (0.62–12.5)	0.18	—	—
Had malaria in the past 6 months	Yes vs. no	2.31 (1.01–5.31)	0.05^*^	2.45 (1.02–5.86)	0.04^*^

*Note*: Variables included in the multivariable model were age, marital status, medical insurance, ever hearing or seeing messages on CC, knowledge of where to get CC services, HIV status, use of antibiotics, and having malaria in the past 6 months.

Abbreviations: aOR, adjusted odds ratio; CC, cervical cancer; CI, confidence interval; cOR, crude odds ratio; HIV, human immuno‐deficiency virus.

* *p* value < 0.05.

## 4 Discussion

Disease screening is crucial for early detection, treatment, and control of health conditions, especially in low‐resource settings. Understanding the factors influencing disease screening allows for targeted interventions tailored to address barriers and preferences among the target population. This study aimed to determine the determinants of CC screening among rural women in Zimbabwe. Our findings revealed that one in three participants had undergone CC screening. While this rate remains low, it marks an improvement from previous studies conducted in Shamva District (2011) and Hurungwe District (2017), Zimbabwe, which reported CC screening rates of 9% (UNAIDS Zimbabwe 2016) and 5% (Fitzpatrick et al. 2020) among rural women, respectively. A nationwide survey in 2015 reported screening rates of 13.4% (Isabirye et al. 2022), and a regional meta‐analysis reported a pooled CC screening uptake of 12.9% (Yimer et al. 2021). The high screening coverage observed in this study can be attributed to an earlier CC self‐sampling study conducted in some of the communities in 2017 (Fitzpatrick et al. 2019). Nevertheless, the current rates remain insufficient for Zimbabwe to meet targets for the WHO's Global Strategy for eliminating CC as a public health problem by 2030, aiming for 70% of women to be screened for CC by age 35 and 45 (American Cancer Society 2024).

Our study identified several factors associated with CC screening uptake. Young age (25–45 years) and lack of medical insurance were associated with reduced odds of CC screening, while exposure to CC screening messages, recent use of antibiotics, HIV‐positive status, and recent malaria diagnosis were associated with increased odds of screening. These were proxies for increased contact with the healthcare system and indirectly revealed the lack of community‐based awareness campaigns. To enhance the effectiveness of CC screening programs, targeting young women will be pivotal for early diagnosis and, thus, timely treatment of cervical dysplasia, aligning with recent WHO guidelines recommending screening start at 25 years for women living with HIV and 30 years for the general population of women (World Health Organization 2021).

Interestingly, our study found reduced screening odds among young women, as noted in other studies (Devarapalli et al. 2018; Yimer et al. 2021). The age eligibility recommendations for screening may lead to complacency among young women, who may perceive themselves as low‐risk. Screening rates may be high among older women because they perceive themselves as a high‐risk group and, therefore, find screening more applicable and beneficial to them (Musonda et al. 2022). Efforts to encourage screening young women living with HIV (25 years) and the general population women (30 years) are essential to prevent late diagnosis.

We also observed increased odds of CC screening among women living with HIV, likely due to the elevated perceived risk of developing CC and increased access to health services within this subgroup. In addition, CC screening is emphasized among women living with HIV. HIV infection amplifies the lifetime risk of HPV infection and inversive CC (Castle, Einstein, and Sahasrabuddhe 2021). In Zimbabwe, the integration of CC screening into HIV care services facilitates access to information and screening because these women are prioritized for CC prevention. Treatment guidelines recommend that women living with HIV should have a Pap smear as they start taking antiretroviral treatment and thus are likely to have ever screened. Screening messages communicated by healthcare workers must be packaged in a way that also attracts HIV‐negative women who may assume CC screening is only for women living with HIV.

Similar to our findings, high screening rates were observed among individuals with medical insurance (Isabirye et al. 2022; Tiruneh et al. 2017), reflecting the toll of economic disparities in accessing healthcare. An estimated 10% of Zimbabweans have health insurance mostly covered through work‐based contributions, and most of those covered pay considerable co‐payments to access health services (Ministry of Health and Child Care 2017). Although the government and its health partners have widespread free CC screening programs, financial concerns remain a barrier to screening. The cost of visiting the facility and other out‐of‐pocket hidden payments in the absence of publicly funded health insurance companies are barriers to using free screening services in Zimbabwe (Mhazo, Maponga, and Mossialos 2023). In addition, fee negotiation impasses between physicians and medical insurance are common, and in such cases, insured individuals are unfairly forced to pay out‐of‐pocket (Mhazo, Maponga, and Mossialos 2023). Addressing this population's economic challenges will be pivotal to promoting screening. A national health insurance program and women's empowerment can enhance access to health services, particularly among the underserved living in rural areas. Increasing and intensifying community screening services brings screening services proximal to the women, thus enhancing the uptake of screening services.

Seeing or hearing CC‐related messages was found to be associated with increased odds of screening uptake. Marketing screening services through simplified messages such as pictorial pamphlets, word of mouth, and community campaigns help to amplify service reach in rural areas. Most rural communities are conservative, and given the discreet nature of CC screening, education messages should be tailored to match the expectations of local culture and accommodate the literacy level of the target population (Yimer et al. 2021). The association between having malaria and being on antibiotics during the last 6 months with increased screening can be explained by increased contact with healthcare workers, hence receiving more information about the importance of screening (Phaiphichit et al. 2022). Healthcare utilization increases the uptake of CC screening, and thus, increasing access to healthcare directly leads to receiving information and addressing misconceptions about the screening programs.

While efforts to improve screening coverage are essential, adequate resources for diagnosis and treatment are equally critical for program success. An organized system linking screened women to care is imperative, emphasizing the need for comprehensive CC control programs (Lopez et al. 2017).

Limitations of this study include its cross‐sectional design, which precludes causal inference, and reliance on self‐reported data, which may be subject to information bias. Additionally, the study's geographic limitation to a rural area may limit the generalizability of findings to the entire country. Using “ever been screened” as the definition for CC screening uptake without accounting for the recommended screening intervals for women may have led to overestimating the proportion of women screened. Nonetheless, these findings provide valuable insights for program planners and implementers to address barriers and enhance CC screening initiatives in Zimbabwe.

## 5 Conclusions

Despite having a high CC disease burden, Zimbabwe faces significant challenges in achieving the WHO target of eliminating CC as a public health problem by 2030. Low CC screening rates among rural women underscore the need to align screening program activities with age, medical insurance coverage, HIV status, and targeted messaging. We recommend strengthening community campaigns, economic empowerment, and healthcare systems to improve CC screening access and reduce the disease burden among underserved rural women.

## 6 Authors Contributions

Study conception and design: Megan Burke Fitzpatrick and Racheal S. Dube Mandishora. Funding acquisition: Megan Burke Fitzpatrick. Data collection: Oscar Tapera, Bothwell Guzha, Megan Burke Fitzpatrick, and Racheal S. Dube Mandishora. Analysis and interpretation of results: Paddington T. Mundagowa and Mufaro Kanyangarara. Draft manuscript preparation: Paddington T. Mundagowa. All authors reviewed and approved the results and final version of the manuscript.

## Ethics Statement

Ethical clearance for this study was obtained from the Medical Research Council of Zimbabwe (MRCZ/A/2621). Permission to conduct the study was sought from the health authorities in the study area, and written informed consent was obtained from all participants.

## Consent

Written informed consent was obtained from all individual participants included in the study.

## Conflicts of Interest

The authors declare no conflicts of interest.

## Data Availability

The datasets used and/or analyzed during the current study are available from the corresponding author upon reasonable request.
